# Dr. Benjamin Van Etten Dolph

**Published:** 1913-05

**Authors:** 


### OBITUARIES.

Dr. Benjamin Van Etten Dolph (not listed in State Directory) died in Sodus, March 26, aged 38.

